# Characterisation of formulated high-density poly(ethylene) by magic angle spinning nuclear magnetic resonance

**DOI:** 10.1039/d4py00010b

**Published:** 2024-03-06

**Authors:** Alyssa M. Rose, Andrew R. McLauchlin, George Wilson, Tom O. McDonald, Frédéric Blanc

**Affiliations:** a Department of Chemistry, Crown Street, University of Liverpool L69 7ZD UK frederic.blanc@liverpool.ac.uk; b Henry Royce Institute, Department of Materials and Department of Chemistry, University of Manchester M13 9PL UK; c Leverhulme Research Centre for Functional Materials Design, Crown Street, University of Liverpool L7 3NY UK; d Stephenson Institute for Renewable Energy, Peach Street, University of Liverpool L69 7ZD UK

## Abstract

High-density poly(ethylene) (HDPE) is an important class of polymer used extensively in plastic packaging as well as numerous other applications. HDPE has a structure that consists of crystalline (monoclinic and orthorhombic) and amorphous domains. Here, we exploit a range of approaches focusing on magic angle spinning (MAS) nuclear magnetic resonance (NMR) aimed at comparing the effect of the HDPE sample formulation (cutting, shaving and cryomilling), from the commercially available manufactured pellets, into these domains and their quantification. ^13^C cross polarisation (CP) experiments reveal that these formulated HDPEs are qualitatively different and ^13^C CP build-up curves and ^13^C direct excitation experiments enable the content of each domain to be obtained, pointing to an increase of monoclinic domain at the expense of the orthorhombic one upon increased processing. The crystallinity contents obtained compared, in some cases, favourably with those obtained by differential scanning calorimetry (DSC) data. These results provide evidence that the manner of preparation of HDPE pellets modifies the concentration of the various domains and suggest that care should be taken during processing.

## Abstract 2

Solid-state ^13^C nuclear magnetic resonance spectroscopy reveals contrasting differences in the concentration of the various domains in formulated high-density poly(ethylene).

## Graphical abstract

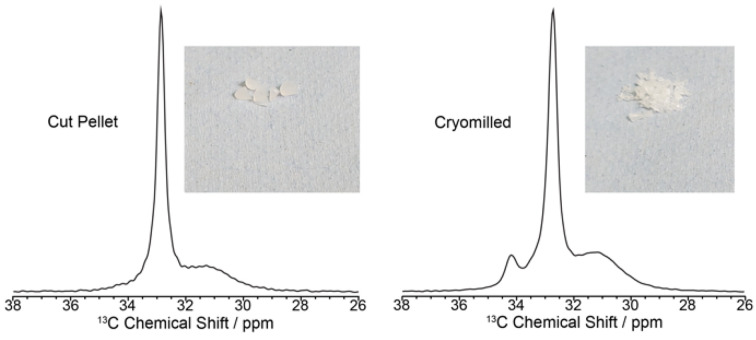

## 1 Introduction

Plastics have become a common commodity in society, with high-density poly(ethylene) (HDPE) being a common plastic used for both food and non-food packaging: 268 thousand tonnes of HDPE were used for packaging in 2017 ^1^ and in accordance with the UK Plastics Pact of 2018, packaging companies have pledged to achieve 30% recycled content in plastics packaging by 2025.^2^ A key to this sustainability goal relies on efficient mechanical recycling technologies processing recyclates containing varying levels of different polymers, grades of plastic and additives/contaminants.^3^ However, recyclates have been shown to vary widely in their properties and performance.^4–6^ This diversity of polymers and additives in post-consumer recycled materials dictates the necessity to have powerful and reliable means of characterizing these materials to understand their structure–property relationship.^7^

Solid-state magic angle spinning (MAS) nuclear magnetic resonance (NMR) is an extremely powerful tool for determining the structural characteristics and dynamics of materials at the local atomic scale and complement well other approaches such as powder X-ray diffraction (powder XRD) and differential scanning calorimetry (DSC) that access long range order and thermal properties, respectively. Particular important features of the NMR approach rely on its non-destructive nature, its quantitativity, the sensitivity of the nuclear spins to the chemical environments (including crystal packing and disorder) and the opportunity to manipulate their behaviours.^8^ However, obtaining high resolution NMR spectra in solids require the removal of the signal broadening arising from the anisotropic NMR interactions which is achieved by spinning the sample at fast rates (typically tens of kHz) at an angle of 54°74′ with respect to the direction of the external magnetic field using MAS.

In order for the sample to spin safely inside the NMR probe and at a constant rate, it is generally accepted to use powdered samples with sub-mm particle sizes. However, the majority of commercially available plastic materials, including HDPE, are manufactured as pellets with a diameter between 4–5 mm; a form that is unsuitable for MAS. Additionally, it can be difficult to process these samples into a pulverized state suitable for MAS, particularly without this pulverization process influencing the properties of the plastic at the atomic scale level.^9^

While standard procedures exist to obtain liquid-state NMR data from sample dissolution^10^ or sample melting directly into an NMR tube,^11^ a few different methods have been considered to prepare HDPE samples suitable for MAS NMR. These include processing the HDPE into fibers^12^ or into sheet subsequently rolled into the rotor,^13^ melting and pressing into a compression-moulded plate,^14^ utilization of a plug the size of the rotor,^15^ cutting or punching out disks the size of the internal diameter of the rotor followed by stacking^16–18^ original pellets^19,20^ and by crushing.^15^ In cases where the width of the pieces of polymer created voids, inert filler was also used to enhance the weight distribution across the rotor.^13,17^ These previous experiments resulted in data that identified the various structural attributes such as phase composition (crystalline, amorphous, interphase, crosslinking), crystal dimensions, molecular mobility, chain branching, deformation mechanisms, optimum methods for the quantification of phases and that probed ways in which these phase structures can be manipulated under various physical conditions.^12,14–20^ However, there seems to be little consensus on the effect of the sample processing preparation methods for high resolution MAS NMR or systematic comparison between those.

In this paper, we explore three different, complementary ways to formulate HDPE pellets for MAS NMR consisting of cutting pellets, shaving and cryomilling them, and assess their effects on the various crystalline (monoclinic and orthorhombic) and amorphous domains observed from ^13^C MAS NMR spectroscopy. While quantification is typically achieved from ^13^C multiple cross polarisation (MultiCP) experiment, the condition for quantitativity^21^ is not achieved in formulated HDPEs, and ^13^C cross polarisation (CP) build-up curves and ^13^C direct excitation spectra have thus been employed for quantification. The data highlight the significant increase in concentration of the monoclinic domain at the expense of the orthorhombic domain with almost retention of overall crystallinity content, which is, in some cases, also captured by DSC with small differences between the formulated HDPE pellets ascribed to the energy of the grinding process.

## 2 Materials and methods

### 2.1 Sample preparation

Virgin HDPE is a Rigidex HD5502S pellet sourced from INEOS which was copolymerized from a low weight percent co-monomer to prepare a medium molecular weight copolymer. The commercial pellets are a bright white color with a circular shape of diameter of roughly 4–5 mm in size measured with a ruler.

Virgin HDPE cut pellets were obtained by manually cutting the commercial pellet into smaller pieces (∼2 mm width) with a stainless steel razor to fit tightly the insert for MAS NMR (see below). Shaved HDPEs were formulated by pressing pellets into a sheet followed by shaving off filaments using a carbon steel file. Cryomilled HDPEs were cryogenically ground using a Spex 6775 Freezer Mill (SPEX Inc, Metuchen, NJ 08840 US). The grinding procedure was: 3 minutes agitation at 13 cycles per second followed by a 3-minute cool cycle, repeated 5 times with liquid nitrogen coolant. This process produced granular samples, much smaller than the pellets, rather than powdered samples.

### 2.2 X-ray diffraction experiments

Powder XRD data were collected in transmission mode on a Panalytical X'Pert PRO MPD equipped with a high throughput screening XYZ stage, X-ray focusing mirror and PIXcel detector, using Cu Kα radiation (*λ* = 1.5406 Å). Data were measured on loose powder samples held on thin Mylar film in aluminium well plates, over the range 4 to 40° in approximately 0.013° steps over 60 minutes. Debye–Scherrer estimation of the coherence length was performed using the following equation1
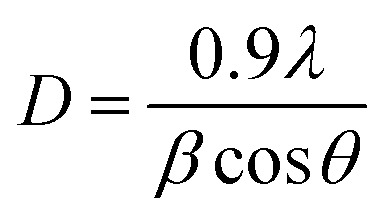
where *D*, *β* and *θ* are the mean coherence length and the full width at half-maximum height (fwhm) and diffraction angles of the reflections. The fwhm was obtained after baseline correction fitting the reflections to a Lorentzian line using a Peak Analysis tool in the Origin™ software package. The peaks corresponding to [110], [200] and [020] planes were used and a consistent average value is reported for each HDPE.

### 2.3 Solid-state NMR experiments

Solid-state NMR experiments were performed under MAS on either a 400 MHz Bruker Avance III HD spectrometer in a 9.4 T magnetic field equipped with a 4 mm HXY MAS probe setup in double resonance mode and tuned to ^1^H at 400.1 MHz and ^13^C at 100.1 MHz, or a 800 MHz Bruker Avance NEO spectrometer in a 18.8 T magnetic field equipped for ^1^H experiments with a 1.3 mm HX MAS probe tuned to ^1^H at 800.3 MHz and for ^13^C experiments with a 3.2 HX MAS probe tuned to ^13^C at 201.2 MHz. To reduce further the risk of rotor crash under MAS in the larger sample volume rotor of 4 mm at 9.4 T, each formulated sample was packed into an Kel-f high-resolution MAS insert then placed into a 4 mm ZrO_2_ rotor and spun slowly at either 3 kHz (cut pellet HDPE) or 4 kHz (shaved and cryomilled HDPEs).

^13^C MAS NMR spectra were obtained at 9.4 T for all HDPEs using ^1^H–^13^C CP and ^13^C direct excitation experiments and were all acquired under ^1^H heteronuclear decoupling using the SPINAL64 scheme^22^ at a radio frequency (rf) field amplitude of 60 kHz. For CP, the ^1^H rf during the 2 ms contact time was 60 kHz and matched experimentally for maximum signal to the ^13^C rf at approximately 56 kHz. The ^1^H 90 degree pulse rf was set to 3.0 μs. ^13^C CP build-up curves were conducted by varying the contact time from 0 to 8 ms with a recycle delay of 7 s using 256 scans. For the ^13^C direct excitation experiments, the ^13^C 90 degree pulse rf was 4.2 μs and recycle delays equal to at least 5 times of the longest ^13^C longitudinal relaxation time *T*_1_ values in the spectrum were used. For the ^13^C *T*_1_ measurements (see below), the ^13^C 90 degree pulse rf was set to 4.2 μs.

The additional ^1^H–^13^C CP spectrum at 18.8 T for the cryomilled HDPE was acquired under MAS at 10 kHz using a standard 3.2 mm ZrO_2_ rotor fully packed and under ^1^H SPINAL64 heteronuclear decoupling at a rf field amplitude of 80 kHz. For CP, the ^1^H rf during the 2 ms contact time was 60 kHz and matched experimentally for maximum signal to the ^13^C rf at 50 kHz. The ^1^H 90 degree pulse rf was set to 3.0 μs.

The ^1^H *T*_1_ was obtained at 9.4 T using a carbon-detected ^1^H saturation recovery experiment in which the saturation recovery experiment is performed first on the ^1^H nucleus with a variable delay of up to 30 s, followed by magnetisation transferred step to ^13^C during a 50 μs CP contact time, and then ^13^C signal detection. The ^13^C *T*_1_ was also obtained at 9.4 T using the Torchia method^23^ with a 2 ms CP contact time and a variable delay of up to 3000 s.

The ^1^H MAS NMR spectra were obtained at 18.8 T under MAS at 55 kHz using a rotor-synchronised Hahn echo sequence (one rotor period). The ^1^H 90 degree pulse rf was set to 3.0 μs.

All ^1^H and ^13^C NMR spectra were referenced to adamantane at a chemical shift of 1.8 ppm (ref. 24) and 29.45 ppm (ref. 25) (for the upfield –CH peak), respectively.

### 2.4 NMR data analysis

NMR data were processed with TopSpin 4.1.3 using standard methods and spectra deconvoluted using the solid lineshape analysis module available. ^1^H *T*_1_ times were obtained by integrating the 1D spectra and fitting the signal amplitudes *I* to an equation of the form2
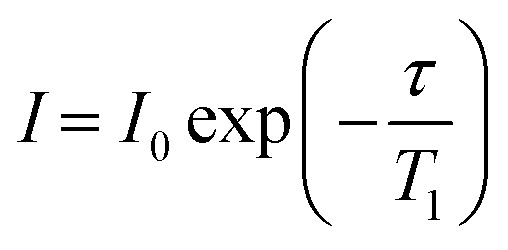
where *τ* is the variable delay. ^13^C *T*_1_ times were obtained by integrating each region of the 1D spectrum and fit the signal amplitudes *I* to an equation of the form3
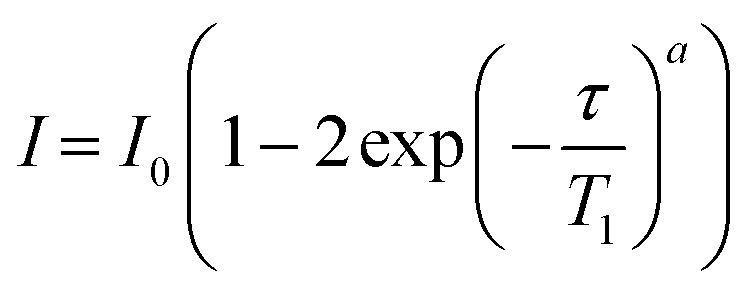
where *a* is the exponential stretch factor. For analysis of the ^13^C CP build-up curves, each spectrum was fitted with the same parameters defining the lineshape and the amplitudes of the signals was varied. These curves were then fitted with MatLab 2023b Curve Fitter app using the simplest model of ^1^H–^13^C CP kinetics (where the ^1^H–^13^C heteronuclear dipolar interactions are relatively weak and ^1^H–^1^H interactions strong providing efficient spin diffusion) using the following equation4
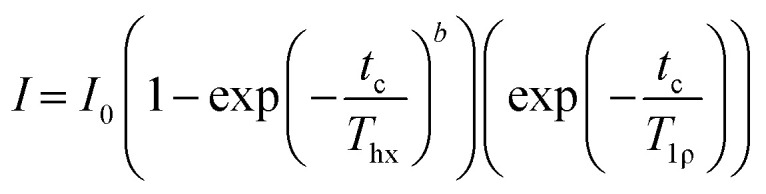
where *I*_0_ is the absolute signal amplitude of the component, *t*_c_ is the CP contact time, *T*_hx_ is the build-up time constant, *b* is the exponential stretch factor and *T*_1ρ_ is the longitudinal relaxation time of ^1^H in the rotating frame. The *I*_0_ obtained from each fitted component is then used to obtain the quantitative fraction of each domain in HDPE. A summary of these fitted values are given below in Table 1.

**Table 1:** Build-up time constant *T*_hx_, exponential stretch factor *b* and ^1^H longitudinal relaxation time *T*_1ρ_ for all domain regions of the formulated HDPE samples

Domains	Samples		
	Cut pellet	Shaved	Cryomilled
Build-up time constant *T*_hx_/μs			
Monoclinic	17 ± 15	30 ± 4	35 ± 5
Interphase	18 ± 14	21 ± 4	40 ± 9
Orthorhombic	63 ± 6	50 ± 7	34 ± 4
Amorphous	190 ± 18	136 ± 22	113 ± 11
Exponential stretch factor *b*			
Monoclinic	0.5 ± 0.3	0.7 ± 0.1	0.6 ± 0.1
Interphase	0.5 ± 0.3	1.3 ± 0.5	0.5 ± 0.1
Orthorhombic	0.5 ± 0.1	0.5 ± 0.1	0.6 ± 0.1
Amorphous	0.7 ± 0.1	0.6 ± 0.1	0.7 ± 0.1
^1^H longitudinal relaxation time *T*_1ρ_/ms			
Monoclinic	11 ± 4	10 ± 1	21 ± 3
Interphase	8 ± 2	9 ± 1	10 ± 1
Orthorhombic	81 ± 30	21 ± 3	78 ± 30
Amorphous	16 ± 2	15 ± 3	17 ± 2

### 2.5 DSC NMR data analysis

All thermal analysis data was collected on a TA Instruments Discovery differential scanning calorimeter. Samples of 5.0 (5) mg were either cut from individual pellets, or weighed from cryomilled pellet as required and sealed in aluminium pans. To determine the melting point and enthalpies of fusion and crystallization, samples were equilibrated at 50 °C under a nitrogen atmosphere, heated to 200 °C, held at temperature for 3 minutes, cooled to 50 °C, held at temperature for 3 minutes and finally heated to 200 °C. The heating and cooling rates were 10 °C min^−1^. The percentage crystallinity was calculated from the normalised enthalpy of fusion (Normalised_HDPE_) by dividing by the heat of fusion of 100% crystalline HDPE (Δ*H*_HDPE_ = 293.6 J g^−1^) using the following equation5
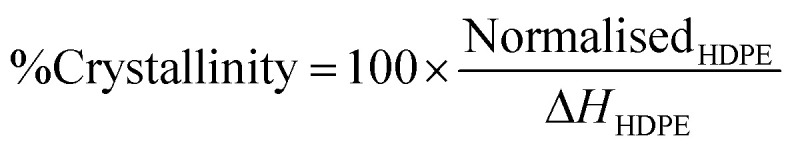


## 3 Results and discussion

**Fig. 1:**
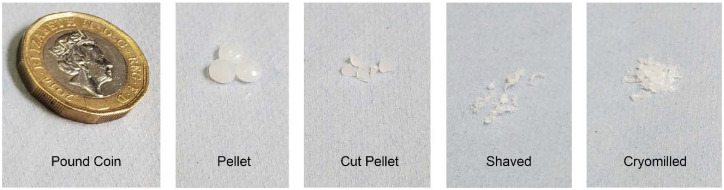
Comparison of the size and shape of the different forms of HDPE; commercial pellet with the three formulated samples (cut pellet, shaved and cryomilled) and a £1 coin for scale.

The virgin HDPE was sourced as large pellets (4–5 mm diameter) as commonly available in the chemical manufacturing sector and were formulated using three different approaches (cut pellet, shaved, and cryomilled, see Materials and methods section for details) suitable for collecting solid-state NMR spectra under MAS. These cut pellet, shaved and cryomilled HDPEs were formulated by manual cutting (2 mm), shaving off filaments (<1 mm) from an extruded sheet, and ground into granules (≤1 mm), respectively, and are visually shown in Fig. 1. These formulated samples are more suitable for use in MAS due to a more uniform weight distribution inside the rotor, also minimizing void space for the shaved and cryomilled samples. With our experimental set up (see Materials and methods section) and under the MAS frequency used at 9.4 T (3–4 kHz), the stability of the MAS was very high (less than 1 Hz fluctuations) for virtually an infinite amount of time. In addition, the NMR linewidths at fwhm for the varying structural phases matched those previously detected in literature, as discussed below, justifying the low MAS used.^26^

**Fig. 2:**
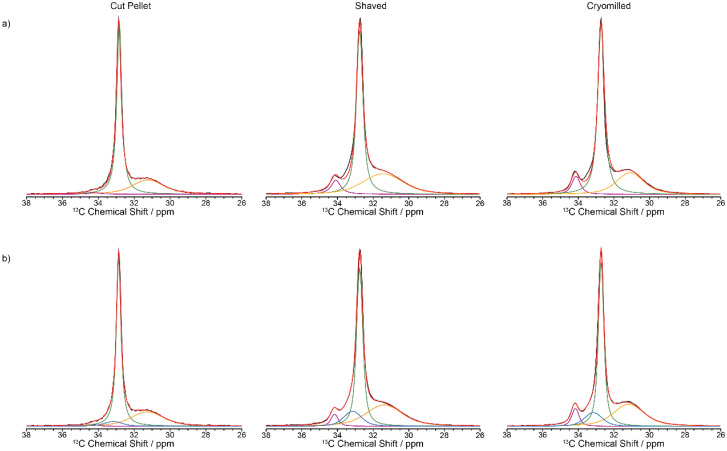
^13^C CP MAS spectra of virgin HDPE cut pellet, shaved and cryomilled samples at 9.4 T with a 2 ms contact time. Experimental spectrum (black), totally fit (red) with (a) 3 components and (b) 4 components are shown (orthorhombic in green, amorphous phase in orange, monoclinic in purple and interphase in blue).

**Fig. 3:**
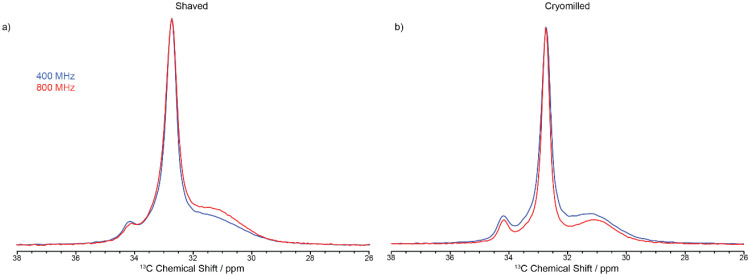
Spectral comparison between CP experiments conducted 9.4 T (blue) and 18.8 T (red) for the (a) shaved samples and (b) cryomilled samples, all collected using a contact time of 2 ms.

The ^13^C CP MAS NMR spectrum of the cut pellet at 9.4 T is given in Fig. 2 and revealed one relatively narrow resonance at 32.7 ppm with a fwhm in the order of 34 Hz alongside a much broader signal centred at 31.2 ppm (fwhm ∼ 186 Hz), broadly in agreement with previous work^26^ (fwhm of 25 and 106 Hz for crystalline and amorphous phases, respectively), and indicate that the slow MAS frequency used is sufficient enough to resolve the characteristic HDPE NMR lineshape. In this polymeric system, the linewidths provide access to the extent of local order and these signals are assigned to orthorhombic and amorphous domains, respectively.^27^ The orthorhombic crystalline phase is the primary crystalline structure in HDPE^28^ consisting of only *trans* confirmations while the amorphous domain arises from disordered region and free motion of the chains leading to both *trans* and *gauche* confirmations.^29^ It is worth pointing out that the presence of *gauche* confirmations in the amorphous region gives rise to its slightly lower ^13^C chemical shift compared to the NMR signal of the crystalline orthorhombic domains.^27^ Upon significant sample processing *via* shaving and cryomilling as detailed in the Materials and methods section, the ^13^C CP MAS NMR spectra are still dominated by these two signals while a third resonance, that is narrow (fwhm ∼ 38 Hz), appears at a higher chemical shift of 34.2 ppm and is attributed to a monoclinic domain.^30^ Attempts to resolve these signals more significantly by recording the ^13^C CP MAS NMR spectrum at higher external magnetic field strength of 18.8 T and faster MAS only resulted in a very marginal improvement in resolution (see Fig. 3).

**Fig. 4:**
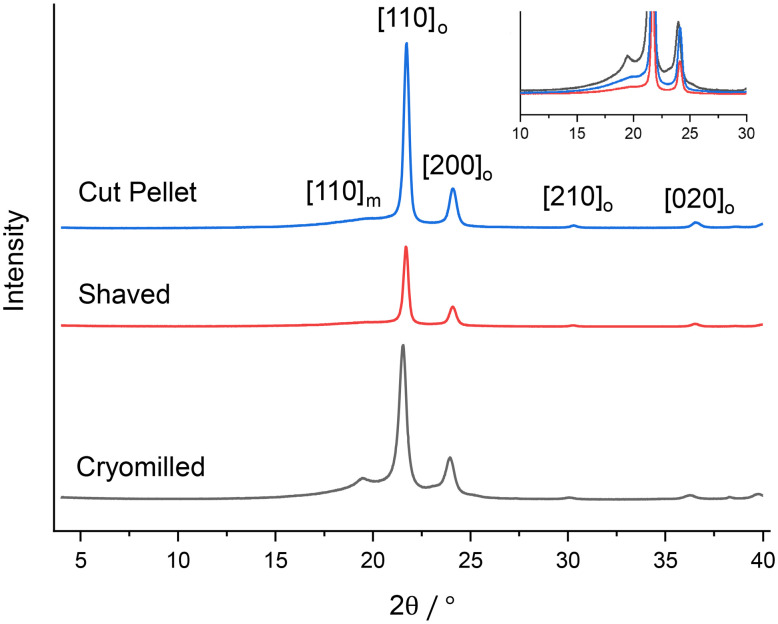
Powder XRD patterns for the cut pellet, shaved and cryomilled samples. The Miller indices of the reflections are given in square brackets where the subscripts *m* and *o* correspond to the monoclinic and orthorhombic phases. The insert shows a magnified view of the amorphous domain in the powder XRD patterns.

The spectra obtained at 9.4 T (Fig. 2) are qualitatively in agreement with the XRD data (Fig. 4) obtained on these samples with the observation of both crystalline phases and an amorphous domain which is more visible in the diffractogram of the cryomilled sample, suggesting that the milling process converted some of the crystalline fraction, possibly due to localized melting under impact. Previous experimental work has shown that the monoclinic domain is formed under stress conditions at the expense of the more stable orthorhombic domain.^31^ This monoclinic domain observation highlights that during processing the samples reached the critical stress value which is then maintained post sample processing. One hypothesis is that the internal stresses present stabilize the monoclinic domain.^18^

Debye–Scherrer analysis has also been conducted on the various HDPE samples. While the coherence length of the crystalline phases in the cut pellet (24 ± 5 nm) and shaved sample (26 ± 5 nm) are similar indicating that shaving has no significant influence on the size, the one of the cryomilled sample (17 ± 4 nm) is significantly smaller. This could be explained in terms of the cryomilling process having a simultaneous heating and cooling effect, thereby melting and rapidly cooling the HDPE so that crystallite growth is arrested.

Close inspection of the spectral deconvolution of the ^13^C CP MAS NMR spectra of the cut pellet and formulated HDPEs (Fig. 2) reveal that the experimental spectra are best fitted with four components (Fig. 2b) rather than three (Fig. 2a), in particular to capture a ^13^C signal contribution in the shaved and cryomilled spectra at a chemical shift centred at 33.2 ppm. This signal is tentatively identified as an interphase region bridging between crystalline and amorphous domains and is based on previous extensive NMR and Raman studies.^14,27,30,32–34^ The interphase region has a fwhm of around 125 Hz and shares spectral similarities with both the crystalline and amorphous phases, with order along a chain and disorder in the lateral direction of the chain, and is considered to be a region with some degree of motion.^14,32–35^ In the current dataset presented in this work (Fig. 2), the line width of the interphase region is broad, similar to the amorphous region, but the chemical shift is similar to that of the crystalline orthorhombic domain. This suggests spectral features of both phases are present in the interphase, such as an ordered amorphous structure consisting of only *trans* confirmations.^27^

**Fig. 5:**
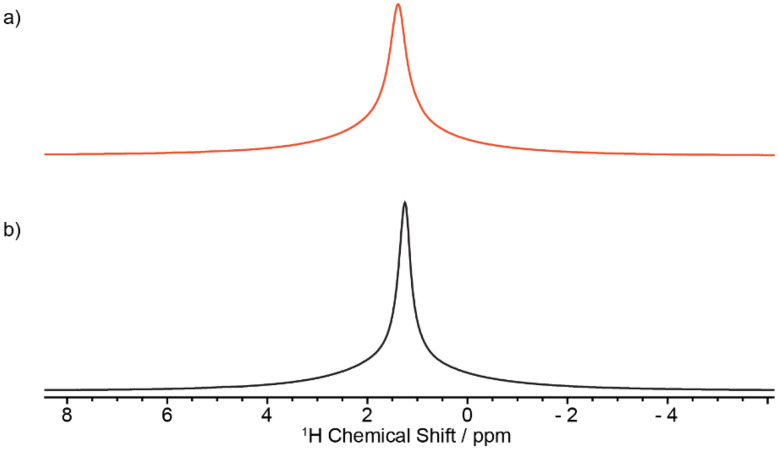
^1^H MAS Hahn-echo spectra of (a) shaved (red) and (b) cryomilled (black) samples obtained at 18.8 T. The ^1^H MAS NMR spectrum of the cut pellet was not recorded due to the larger size of the pellet than the 1.3 mm rotor used.

Several NMR approaches could be used to quantify each structural component and are either based on multiple cross polarisation (multiCP),^21,36^^13^C CP as a function of contact times (CP build-up curves) or quantitative ^13^C polarisation experiments. Each of those experiments has advantages and drawbacks. Direct polarisation is arguably the most straightforward and is quantitative with adequately long recycle delays which this is therefore an important drawback given that these delays often result in time-consuming experimental time (typically days to collect spectra with significant signal-to-noise ratio, SNR). CP results in spectra that can be collected much more rapidly than direct polarisation and with very good SNR, but the resulting spectra are not quantitative and a CP build-up curve (that is signal intensity *vs.* contact time) is needed and fitted to a model (see eqn 4 in Materials and methods). MultiCP is a variant of the CP experiment that yields quantitative MAS spectra with high SNR and relies on repeated blocks of CP steps separated by periods of ^1^H repolarisation to near equilibrium during which there is minimum ^13^C loss of polarisation, thereby requiring ^13^C *T*_1_s to be significantly longer than ^1^H *T*_1_s. Although multiCP would offer an efficient approach for quantitative MAS spectra and this condition mentioned above is very often met in organic solids, it was found that this is not the case for HDPE, likely due to the various existing domains and perhaps the heterogeneity of the samples. The ^13^C *T*_1_s (measured using a CP inversion recovery experiment, as described in the Materials and methods section) displayed significant variations for the various signals observed and, as expected, the ^13^C *T*_1_ values of the two crystalline domains (on the order of hundreds of seconds, Table 2) are significantly longer than ^13^C *T*_1_ of the amorphous domain (below 1 s, Table 2) highlighting the increased mobility in this region.^32^ The ^1^H *T*_1_s were measured using a carbon-detected ^1^H saturation recovery experiment (as described in the Materials and methods section) and are broadly similar (about 1 s, Table 2) for all domains for all formulated HDPEs likely due to the strong ^1^H–^1^H homonuclear dipolar coupling that dominates the lineshape (Fig. 5) and homogenizes *T*_1_ relaxation. Most importantly, the ^13^C *T*_1_ of the amorphous phase is in the same order of magnitude than that of the ^1^H (Table 2), rendering the multiCP approach unsuitable for the quantification of all domains in these heterogeneous systems.

**Table 2:** ^13^C and ^1^H isotropic chemical shifts *δ*_iso_ and relaxation times *T*_1_ for all domain regions of the formulated HDPE samples

Domains	*δ*_iso_/ppm^a^	Samples		
		Cut pellet	Shaved	Cryomilled
		*T*_1_/s		
	^13^C			
Monoclinic	34.2	1200 ± 590	460 ± 190	550 ± 110
Interphase	33.2	—b	—b	—b
Orthorhombic	32.7	930 ± 130	800 ± 100	700 ± 100
Amorphous	31.2	0.032 ± 10^c^	0.29 ± 5^c^	0.37 ± 4^c^
	^1^H			
Monoclinic	1.3	1.4 ± 0.7	1.3 ± 0.8	1.4 ± 0.3
Interphase	1.3	—b	—b	—b
Orthorhombic	1.3	1.2 ± 0.2	1.3 ± 0.1	1.2 ± 0.1
Amorphous	1.3	1.3 ± 1.2	1.1 ± 0.5	1.4 ± 0.4

a Given with 0.2 and 0.5 ppm accuracy for ^13^C and ^1^H, respectively.

b Not determined due to a lack of resolution in the ^13^C CP MAS NMR spectra.

c Significant error bars likely due to the fast decay of this signal, further supporting that these *T*_1_s are much shorter.

Fig. 3 shows the ^13^C CP MAS NMR spectra of the shaved and cryomilled samples at 9.4 T with those at 18.8 T with faster MAS rate and stronger ^1^H decoupling amplitude (see Materials and methods section for all relevant details). Note that a ^13^C CP spectrum of the cut pellet sample was not run at 18.8 T because of HDPE size restriction to fit into the 3.2 mm rotor used. While a very small increase in resolution is observed at 18.8 T, this is rather visually marginal and justify that higher field, faster MAS and stronger ^1^H decoupling are not necessarily required to achieve an optimum resolution in these polymers. It also highlights that whilst it would be desirable to obtain ^13^C CP MAS NMR spectra with improved resolution, for example to separate the interphase signal from those of the crystalline phases that are separated by a maximum of 1 ppm (Table 2), high field only offers a negligible gain and data at 9.4 T provides sufficient resolution.

**Fig. 6:**
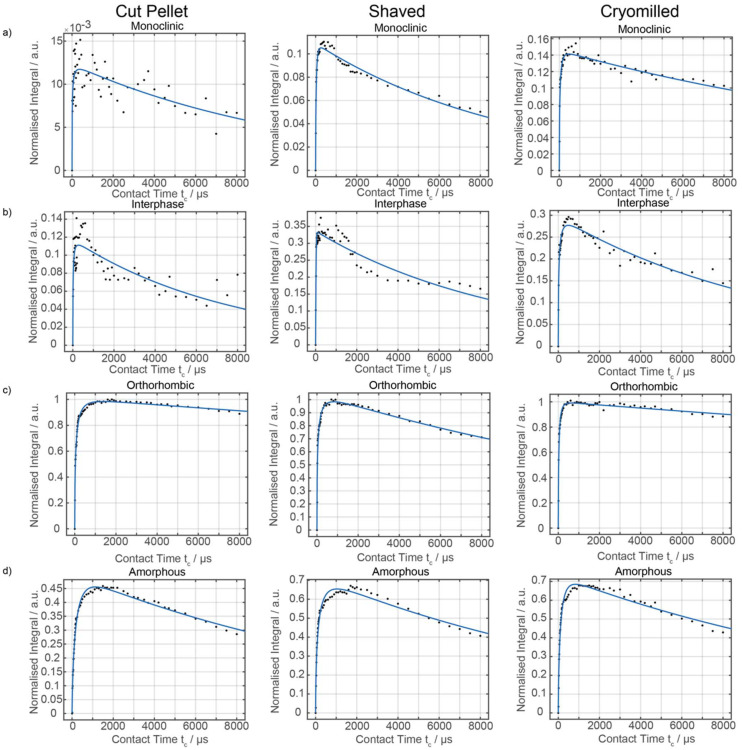
^13^C CP build-up curves for the virgin HDPE cut pellet, shaved and cryomilled samples at 9.4 T for all four domains of the (a) monoclinic, (b) Interphase (c) orthorhombic and (d) amorphous regions. Experimental data points are given in black circles fitted to the simplest CP kinetics model (blue curve) as described in the Materials and methods section and are normalised against the most intense signals of the orthorhombic domain.

Given that ^13^C multiCP is not directly applicable, the two other approaches of ^13^C CP build-up curves and ^13^C direct excitation were then exploited and compared to quantify the various domains in the three formulated HDPE samples, noting that as expected the overall experimental time is significantly longer for ^13^C direct excitation (several days/sample) that for the CP build-up (approximately 1.5 days/sample). ^13^C CP build-up curves were obtained from signal integrals (of all signals as per spectral deconvolution) of a series of ^13^C CP spectra as a function of CP contact times (Fig. 6) for all samples, with the data fitted to a basic CP kinetics model as described in the Materials and methods section. The absolute signal amplitude *I*_0_ is used to obtain the fraction of each domain in the HDPEs sample and is the fitted parameter used for the data quantitation from the CP build-up curves. Other fitted parameters for the model are given in Table 1 and indicate, for example, in the orthorhombic phase, shorter ^1^H *T*_1ρ_ for the shaved sample likely capturing the disorder from additional processing with even shorter values (few ms) for the interphase region. The CP time constant *T*_hx_ which describes how quickly the magnetisation is transferred between ^1^H and ^13^C is found to be the longest in the amorphous region, likely from decreased packing density.

**Fig. 7:**
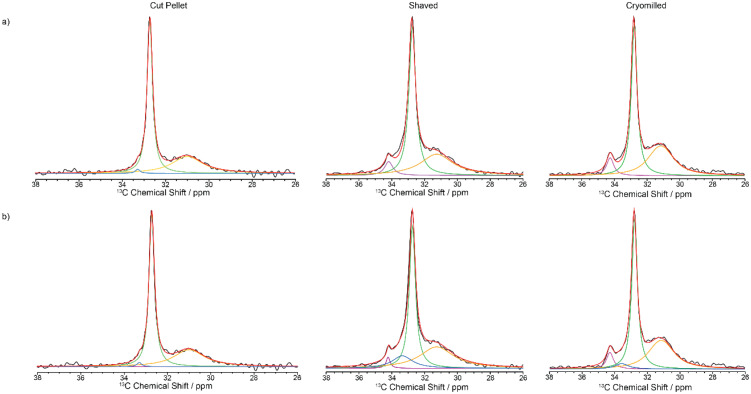
^13^C directly excited spectra of virgin HDPE cut pellet, shaved and cryomilled samples at 9.4 T. Experimental spectrum (black), totally fit (red) with (a) 3 components and (b) 4 components are shown (orthorhombic in green, amorphous phase in orange, monoclinic in purple and interphase in blue).

Whilst the fit is satisfactorily, the discrepancy observed between the experimental data and model likely arises from both the simple model used and the challenge associated with the limited resolution obtained (in particular for the interphase) but capture with some degree the contribution of each domain in these HDPEs. The ^13^C MAS directly excited spectra (Fig. 7) were obtained under standard quantitative conditions with recycle delays longer than five times the longest ^13^C *T*_1_s and are best deconvoluted with four components as discussed above, noting that the monoclinic domain is likely below the sensitivity limit for the cut pellet and thus not observed experimentally.

The absolute amplitudes which relative percentage quantifying the concentration of each domain in the HDPE samples are given in Table 3 for the two quantification NMR methods. The data from the directly excited ^13^C MAS NMR spectra have a much larger margin of error than in the ^13^C CP build-up data (Table 3), which is ascribed to the much lower signal to noise ratio in those spectra. The fractions of the various domains according to these two NMR quantification methods agree within the margin of error. The NMR data reveal that the percentage of the monoclinic domain is the lowest in the cut pellet sample (approx. 1% and below) while both cryomilled and shaved samples contain up to 7% of monoclinic domain as also observed qualitatively in the ^13^C CP MAS spectra in Fig. 2. Additionally, the cut pellet also contains the highest fraction of orthorhombic domain (around 60%) out of all the samples with the cryomilled and shaved samples accommodating around 46–51%. This indicates that the most stable orthorhombic domain is being converted into monoclinic domain during processing as observed in previous work.^31^ It is postulated that these cryomilled and shaved samples have been the most modified by the energy intensive processes that occur during their processing, because monoclinic domains are mostly formed under stress conditions. In this case, it is hypothesized that mechanical stress was induced in the material during the grinding or shaving processes resulting in the introduction of defects into the crystalline phases. Indeed, compression has been shown to induce transformation of HDPE from orthorhombic to monoclinic.^37^ This hypothesis is supported by the change in ^13^C *T*_1_ values observed between the monoclinic and orthorhombic crystalline phases from the cut pellet sample and both processed samples. As given in Table 2, the ^13^C *T*_1_ values for both crystalline regions shorten after processing and could potentially indicate slightly increased mobility due to the introduction of stress-induced defects. The amorphous fraction domain is approximately 35% and is similar between all samples (within error bars) regardless of the processing processes and suggest that this domain is formed intrinsically during the preparation of virgin HDPE. This fraction is comparable with those in the literature (41%^18^–38%^26^ from ^13^C NMR for ultra-high molecular weight HDPE fibers^18^ and with a different morphology,^26^ 12% from ^1^H wideline NMR of ultra-high molecular weight HDPE fibers,^27^ 21% from integrating powder XRD patterns,^38^ 26%^16^ and 47%^26^ from DSC, and 20%^39^ from both IR and SAXS^39^) using a range of approaches on a range of different HDPEs. The interphase domain is present mostly in the shaved and cryomilled samples which is an indication that this domain is also formed and grows with the amount of processing and stress, in agreement with previous data where the interphase component decreased upon annealing.^14^ The domain sizes for the monoclinic and orthorhombic phases have been calculated from the diffusion path length6
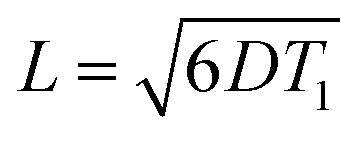
where *D* is the ^1^H spin diffusion coefficient (10^−12^ cm^2^ s^−1^).^40^ The ^1^H T_1_ values were all very similar around 1 s (Table 2), regardless of domain or sample preparation methods, resulting in domain sizes between 25–30 nm which are broadly in agreement with the powder XRD data.

**Table 3:** Percentage of each domain of the different forms of the HDPE samples obtained from two quantitative MAS NMR methods of ^13^C CP build-up curves and direct ^13^C excitation^a^

Domains	Quantification method	Samples		
		Cut pellet	Shaved	Cryomilled
Monoclinic	CP build-up	1.0 ± 0.1	4.9 ± 0.1	6.7 ± 0.2
	Direct Exc.	—b	2.4 ± 9^c^	6.3 ± 10^c^
Interphase	CP build-up	7.0 ± 0.7	15.2 ± 0.6	13.6 ± 0.6
	Direct Exc.	1.4 ± 8.2^c^	13.4 ± 2.0	5.4 ± 11^c^
Orthorhombic	CP build-up	61 ± 2	47.2 ± 2.0	46 ± 1
	Direct Exc.	61.2 ± 1.2	45.9 ± 0.5	51.3 ± 1.0
Amorphous	CP build-up	31 ± 1	32.7 ± 2.0	33.6 ± 1.1
	Direct Exc.	37.4 ± 10.1^c^	38.3 ± 8.0	37 ± 7

a Percentage normalized to 100%.

b Not detected under those experimental conditions.

c Significant error bars likely due to the small signal intensity or poor spectral resolution for the specific resonance.

DSC is commonly used to estimate the percentage of crystalline material in a mixture, by comparing the enthalpy of fusion with the calculated enthalpy for 100% crystalline polyethylene,^41^ as described in the Materials and methods section, and those data are thus compared with the NMR ones presented above (Table 4). First, the crystallinity contents obtained from DSC from the 1^st^ heat endotherm are lower than those obtained from the 2^nd^ heat. This is commonly found to be the case for materials not being fully crystallized and is a consequence of its thermal history. During the heat-cool-heat process, the polymer is annealed and so the crystallinity obtained from the second heat represents the maximum crystallinity for that material. This annealing process is however absent from the thermal history of the MAS NMR data and those percentages are thus compared below with the 1^st^ heat endotherm.

**Table 4:** Comparison of crystallinity percentages obtained from DSC and NMR of the formulated HDPE samples

Quantification method		Samples		
		Cut pellet	Shaved	Cryomilled
DSC	1^st^ heat	59.0 ± 0.8	58.0 ± 3.3	54.4 ± 1.2
	2^nd^ heat	64.2 ± 1.2	63.7 ± 3.6	62.5 ± 1.4
NMR^a^	CP Build-up	62 ± 2	52.1 ± 1.7	52.7 ± 1.1
	Direct Exc.	61.2 ± 1.2^b^	48.3 ± 9.4^c^	57.6 ± 9.9^c^

a Values obtained by summing all crystalline domains only (the amorphous and interphase domains are thus excluded).

b The monoclinic component is not included as not observed under those experimental conditions (see Table 3).

c Significant error bars likely due to the small signal intensity or poor spectral resolution for the specific resonance (see Table 3).

Secondly, the percentage of crystalline materials obtained from both MAS NMR methods are in excellent agreement with each other. Both NMR methods follow the same trend with the concentration of crystalline domains being the lowest in the shaved HDPEs and largest in the cut pellet. However, it is interesting to note that the latter observation is not consistent with the data determined from any of the two thermal treatments in the DSC data, which is not entirely surprising given previous literature highlighting the more accurate NMR approach.^16^ It is also postulated that partial melting due to friction during milling and subsequently partial quenching at the cryogenic temperature during the process is likely responsible for the formation of a lower concentration of crystalline domains in the cryomilled HDPE, a feature which was confirmed in the XRD diffractogram that shows a larger amorphous domain component for this materials (see Fig. 4). Finally, while the crystallinity content in the shaved HDPE falls between those for both cut pellet and cryomilled HDPE in the DSC data, this content of the shaved HDPE is significantly lower in both ^13^C MAS NMR methods and may reflect the larger interfacial domain of the shaved sample.

## 4 Conclusion

There are challenges with processing methods (cut and formulated samples from shaving or cryomilling) used to prepare ground samples from pellets of virgin HDPE for MAS NMR spectroscopy which we show have a significant impact on the domains formed. Several differences were observed by quantitative ^13^C MAS NMR between the pellet and formulated samples both in terms of the content of the various structurally different domains and of the overall crystalline or amorphous domains, some of which are accurately reproduced by DSC measurements. This work illustrates that care should be taken in the manipulation of virgin HDPE pellets as any formulation will alter the concentration of the various domains. These findings also highlight the importance of complementary approaches combining powder XRD, DSC and NMR spectroscopy on multicomponent systems with a range of crystalline and amorphous domains. Given the crucial role of crystallinity in governing the properties of plastics like HDPE, delving into the specific crystal form within the plastic can illuminate how processing parameters influence its characteristics. This characterization approach holds promise for future applications, such as linking the variations in crystalline structure across different regions of plastic items, like bottles, to potential failure mechanisms, such as environmental stress cracking.

## Data availability

The research data supporting this publication are accessible from the University of Liverpool Data catalogue available at https://doi.org/10.17638/datacat.liverpool.ac.uk/2623.

## Conflicts of interest

The authors declare no competing financial interest.

## Supplementary Material
